# Evaluation of the implementation outcomes of the Essential Medicines System in Anhui county-level public hospitals: a before-and-after study

**DOI:** 10.1186/s12913-015-1073-z

**Published:** 2015-09-22

**Authors:** Shuman Xu, Cheng Bian, Heng Wang, Niannian Li, Jingya Wu, Peng Li, Hua Lu

**Affiliations:** Guangdong Women and Children Hospital, 521 Xingnan Road, Guangzhou, Guangdong 511442 P.R. China; The First Affiliated Hospital of Anhui Medical University, 218 Jixi Road, Hefei, Anhui 230022 P.R. China; The Fourth Affiliated Hospital of Anhui Medical University, 372 Tunxi Road, Hefei, Anhui 230000 P.R. China; Anhui Provincial Hospital, 17 Lujiang Road, Hefei, Anhui 230001 P.R. China

**Keywords:** Essential Medicines System, County-level public hospitals, Implementation outcomes, Evaluation

## Abstract

**Background:**

In August 2009, China formally established the National Essential Medicines System (NEMS) and implemented this system in the government-funded primary care medical and health institutions. After nearly four years of practice, the system has already been generalized to the county-level public hospitals. This study aimed to examine the impact on the operation of the hospitals through implementing the NEMS in Anhui Province and put forward some improvement measures.

**Methods:**

For quantitative analyses, we distributed 21 questionnaires to 21 county-level public hospitals in Anhui Province, which had implemented the national public hospital reform. Twenty valid questionnaires were returned, response rate was 95.2 %. Questions covered storage, usage and supply of essential medicines, compensation mechanisms, insurance policies, hospital incomes, service amounts and fees from January to June in each of the years from 2011 to 2013. For qualitative study, we chose three from 21 hospitals based on geographical distribution and conducted focus group interviews based on a planned interview outline centered on the implementation status of the system.

**Results:**

Following implementation, the types of essential medicines stocked and the proportion of total sales that were composed of essential medicines have increased but do not yet meet the required standards issued in the government document, which was not less than 95 % and 30 % of the total, respectively. The average financial subsidies had increased by 1,665,200 yuan, and significant increases appeared in provincial financial assistance. The average inpatient fees per visit decreased by 487.41 yuan. Increases in income from medicines during hospitalization led to increases in per-visit hospitalization fees. Unexpectedly, higher financial assistance revenue also led to higher average per-visit hospitalization fees.

**Discussion:**

The guiding role of the National Essential Medicines List remains to be reinforced, and specific lists for county hospitals should be developed. Supervision was required to implement the process of guaranteeing the storage and usage of essential medicines. The compensation mechanism was far from sound, and the leverage of the health insurance policies was not obvious. Regarding the reductions in the proportion of income derived from medicines and per-visit inpatient fees, the policy had been partially successful.

**Conclusions:**

Our results showed that the implementation of the Essential Medicines System do have a beneficial role in the reduction of the drug fees and further alleviates the burden of the masses. Much effort should be made to the redesign of the compensation mechanism, mainly including the government and the medical insurance compensation, emphasizing on both the fairness and the rationality of the compensation in the future.

## Background

As one of the important health resources, drugs are always related closely with the health quality and safety. Essential medicines, first proposed by the World Health Organization (WHO) in 1977, were defined as “of utmost importance, basic, indispensable and necessary for the health and needs of the population” and criteria relating to safety, quality, efficacy and affordable [1]. Through putting forward the Essential Medicine policy, the WHO intended to promote equitable access to medicines and improving the health of the entire population. In 2008, 42.7 % of China’s total health expenditure was on drugs, much higher than the 17 % for the nations of the Organization for Economic Co-operation and Development [2, 3]. In an attempt to alleviate the burden of drug costs on the population, China formally established the National Essential Medicines System in August 2009. All of the government-funded primary care medical and health institutions in 31 provinces (autonomous regions or municipalities) and the Xinjiang Production and Construction Corps were allocated essential medicines and instituted a “zero mark-up” (i.e., no profit) policy for essential medicines sold after October 2011 according to statistics from the National Health and Family Planning Commission of the People’s Republic of China [4]. When the initial stages of this program demonstrated favorable results, the policy was fully implemented across all medical institutions.

County-level public hospitals typically act as secondary-level general hospitals and belong to a higher level than do the grassroots medical institutions, and these hospitals play a more vital part in the Chinese healthcare system. China began a program of comprehensive reform of these hospitals in June 2012 that created suitable conditions for the implementation of the Essential Medicines System. Recent research had mostly focused on the implementation statuses of the system in primary medical and health institutions, or conducted investigations into the doctors’ prescribing behaviors and rational drug use [5–8]. Little attention has been given to scientific and systematic evaluations that examine the effects of policy and objectively analyze the contributing factors in the county hospitals. The Anhui Province is located in eastern China, covers 74 counties (including cities and districts) and has a population of approximately 70 million. According to the Anhui Provincial Health Department statistics, a total of 23,278 medical and health institutions of all types [9] and 222283 beds [10] were available by 2012. On average, there were only 3.22 beds per thousand of the population [11]. As this populous province has a concerning medical status, the critical and urgent issue is how to protect the quality and level of the medical service and ease the financial burden on the population. Under the new medical reforms, Anhui has continued to explore new directions and is consistently at the forefront of national reforms. Specifically, when the Essential Medicines System was first implemented, some areas of Anhui were the first to adopt the “zero mark-up” policy and tried many innovative approaches to bidding for and purchasing essential medicines. Several of the reforms began in Anhui and then expanded elsewhere. Therefore, we chose Anhui as the research site because there was a greater likelihood for the policy outcomes to be more effective and remarkable. The present study compared the changes in various indices related to the outcomes of the Essential Medicines System and subsequently explored the key factors that influenced the county hospitals’ pre- and post-implementations in the Anhui Province to better identify the existing problems, promote policy and develop hospitals.

## Methods

### Data collection

The National County-level Public Hospital Reform in China has been carried out in Anhui Province since 2012, and 21 counties in total in Anhui were assigned as experimental units at the first round. Using a random sampling method, one county-level public hospital was selected from each of the 21 counties as the quantitative study objects. According to the regional distribution, 21 hospitals were divided into three groups which were group of northern, central and southern Anhui. Then one county-level public hospital was selected from each group as the research sites of qualitative interviews. Totally, 21 county-level public hospitals were involved in quantitative study and 3 in qualitative interviews.

The 21 hospitals primarily implemented the Essential Medicines System in the last three months of 2012. Our study was conducted from July to September in 2013.

The Ethics Committee of the First Affiliated Hospital of Anhui Medical University approved the project (PJ 2014-09-09). Ethical permission for this study was not necessary under Chinese law as no patient data was collected. All the surveyed hospitals had been informed of the investigation in advance and consented to be our participants.

### Qualitative study

The qualitative study was mainly centered on the implementation status of the Essential Medicines System and existing problems with supporting policies, such as the compensation mechanism and medical insurance system, the impact on the medical staff, etc. Focus group interviews were performed in the three selected hospitals via the organization of the government departments. Our investigator was responsible for sound and manual recordings.

### Quantitative study

The quantitative study focused mainly on the implementation outcomes of the Essential Medicines System, reflected in the operation conditions of the hospitals. The standard scale employed by the Anhui Provincial Health Department to evaluate the effects of the medical reform on the county-level public hospitals was a good reference for the questionnaire design. In combination with a literature review, we identified the final indicators and the whole questionnaire. The reliability and validity of the indicators were then reasonably confirmed via consultation of experts. For the core indicators investigated, we covered the periods between January and June for each of the three years from 2011 to 2013. The core indicators of the study can be found in Table 1.

**Table 1:** The main aspects and core indicators of the quantitative study

Main aspects	Core indicators
Storage, usage and supply of essential medicines in the hospitals	The numbers stocked and proportion of drugs sales what were essential medicines, supply delay time and reason
The implementation status of the supporting policies (compensation mechanisms and medical insurance policies)	The means of financial assistance, the financial subsidies at all levels; total cost of medical insurance compensation
Effects on the operation of the hospitals of implementing the policy (i.e., the hospitals’ incomes, service amounts and fees)	Drug income, proportion of income from medicine sales; outpatient and emergency visits, inpatient visits; the outpatient and per-visit hospitalization fees

Communications and coordination regarding the main purposes and contents of this study were performed with the investigated hospitals in advance by the government departments. After clear explanations, the questionnaires were sent as emails to the chiefs of the medical departments who were in charge of the completing the questionnaires in each hospital. Furthermore, a specific time limit was given, after which the investigator contacted the chief by telephone to recover the questionnaires to facilitate high response rates to the fullest extent possible. Ultimately, we obtained 20 valid questionnaires and failed to obtain only one questionnaire.

### Quality control

Prior to the survey, we explained to the hospitals that the survey data would not be used as evaluation or assessment criteria for any of the hospitals or for official purposes, but only for research analysis. We further explained that the questionnaires could be completed anonymously. The concerns of the hospitals were possibly eliminated by this procedure to guarantee the authenticity of the data.

When the questionnaires were returned, two research group members who did not participate in the entire investigation process examined the questionnaires to determine the quality of the data. When any logical error, filling error or instance of incomplete content was found, timely feedback was given to the investigators, who were responsible for subsequent amendments and supplements, to verify the accuracy of the data.

### Data analyses

#### Qualitative data

Word frequency analyses were applied to the qualitative data. We transcribed all of the sound recordings and the manual recordings to establish an interview text database through information summaries and proofreading. All of the relevant keywords, which were classified according to items such as reasons and results, were extracted from the database according to the goals of the interview. Next, the frequencies with which the keywords appeared in the database were counted. If a keyword occurred repeatedly, the problems represented by that keyword may have been important or required urgent resolution.

#### Quantitative data

With the purposes of conducting before-and-after comparisons, maintaining the stability of the data and more clearly highlighting the differences, we averaged the survey data from 2011 to 2012 as the pre-implementation data and compared these data to the post-implementation data from 2013.

All data were entered into Epidata 3.1 twice to ensure accuracy. Statistical analyses were conducted using SPSS (version 16.0). We calculated the means and variance for the quantitative data. The main method was comparative analysis, and statistical significance was assessed using t tests. Single factor analyses of the influences on service fees were performed via correlation analyses followed by multiple linear regression for the multivariate analysis. The level of significance was set at *p* ≤ 0.05.

## Results

### Qualitative results

In total, we conducted three focus group interviews in each of three hospitals, and the durations of these interviews were approximately two hours. The interview participants included officials from government departments, experts in healthcare and hospital management, leaders of the surveyed hospital, chiefs of the medical, pharmacy, finance and other relevant departments of the hospital, doctors’ representatives and trained investigators.

For all three hospitals, it was generally the case that, although the documents regarding the implementation of the Essential Medicines System had been put forward by the government, no further specific implementation regulations or instructions were subsequently formulated, which consequently resulted in the hospitals making their own explorative attempts. It was also common that the main basis of drug procurement and usage for the county-level public hospitals was the previously established “Basic Drugs Directory for County Hospitals” of the government of Anhui Province, which contains all 307 drugs from the National Essential Medicines List of 2009, 276 supplemental essential medicines of the Anhui Province and another 465 non-essential medicines. The directory had not yet been updated after the introduction of the new National Essential Medicines List in March 2013.

After the implementation of the Essential Medicines System, the income sources of the hospitals, which formerly consisted of financial assistance income, business income and drug price addition revenue, were limited to only the financial assistance income and business income. One of the hospitals indicated that the financial assistance was insufficient to make up for the losses of drug price addition revenue. As a result, the hospital found development and even survival difficult. Moreover, the job positivity of the doctors was partially reduced. In contrast, another hospital noted that the existing compensation method of the medical insurance funds for the hospitals was quite unsustainable and that the financial assistance was sufficient.

It could be learned from the interviews that regional disparities existed between the hospitals. However, overall, the issues were mainly concentrated on the supporting policies of the Essential Medicines System.

## Quantitative results

1. The implementation outcomes of the Essential Medicines System in the county-level public hospitals
2. Storage, usage and supply of essential medicines in the hospitals

Prior to the implementation of the Essential Medicines System, the average number of different types of essential medicines maintained in the stocks of the hospitals was 124, and the average proportion of sales that were essential medicines was 9.74 %. After implementation, the average number of stocked essential medicines increased significantly to 250, and the average proportion of sales that were essential medicines increased to 19.4 %.

In terms of the supply of essential medicines, the average delivery delay was 5.5 days. The delays were much shorter for seven hospitals and ranged from two to three days. The delays were four to five days at eight hospitals and much longer in the other five hospitals, ranging from seven to 15 days. These delays were due to the low profits available to the distributing companies, which made them reluctant to distribute. Elsewhere, distribution companies had limited capabilities, or delays were reported of manufacturing hold-ups. It was also possible that traffic, weather and other objective factors affected supply.

1.2 The implementation statuses of the supporting policies (compensation mechanism and medical insurance policy)
1.2.1 Changes to the financial subsidy income before and after implementation

We surveyed financial assistance before and after the implementation of the Essential Medicines System including all levels of financial subsidies and the total grants. The results are displayed in Table 2.

**Table 2:** The financial subsidies of the county-level public hospitals across all levels before and after the implementation of the Essential Medicines System

County-level hospitals	Before implementation				After implementation			
	Total grants (10 thousand yuan)	County (district) (%)	Province (%)	Central (%)	Total grants (10 thousand yuan)	County (district) (%)	Province (%)	Central (%)
A	957.15	100	0	0	1378.7	100	0	0
B	127.29	100	0	0	309.03	100	0	0
C	88	100	0	0	700	0	28.6	71.4
D	177.75	100	0	0	70	100	0	0
E	100	0	100	0	539	34.7	65.3	0
F	197.5	100	0	0	264	72.7	27.3	0
G	74.86	100	0	0	74.92	68.0	32.0	0
H	399	39.9	42.6	17.5	456	59.9	40.1	0
I	470.44	100	0	0	401.38	84.9	15.1	0
J	125	100	0	0	490	73.3	26.7	0
K	236.18	100	0	0	320	100	0	0
L	65.38	100	0	0	680.83	63.3	36.7	0
M	5.28	100	0	0	124.1	16.2	83.8	0
N	37.5	100	0	0	351	69.5	30.5	0
O	151.03	100	0	0	210.56	100	0	0
P	175.88	100	0	0	112.3	100	0	0
Q	363.83	100	0	0	362.83	100	0	0
R	16.2	100	0	0	12	100	0	0
S	335.5	100	0	0	400	100	0	0
T	62.5	100	0	0	240	37.5	62.5	0

We do not list the municipal financial assistances in the table because none of the hospitals received municipal financial assistance before or after implementation

In order of high to low, the levels of the financial subsidies were the central, province, municipal and county subsidies. Before implementation, most of the hospitals were assisted only by the county finance subsidies, although one hospital received all of its assistance at provincial level and another obtained financial assistance from the central, provincial and county governments. None of the hospitals received municipal financial assistance. After implementation, the strength of the financial assistance increased significantly, primarily at the provincial level. Eleven county hospitals had access to financial assistance from the province and county. The proportions of provincial financial assistance ranged from 15.1 % to 83.8 %, which represented a large change from the pre-implementation situation. However, the central and municipal financial assistance efforts were still quite weak or non-existent.

To further understand the specific differences in the levels of financial assistance, we compared the relevant statistical data about the total financial assistance for all hospitals. Before implementation, the income from financial assistance across the 20 hospitals averaged 2,083,100 yuan, and specific grant funds ranged from 52,800 to 9,571,500 yuan. After implementation, financial assistance income averaged 3,748,300 yuan, and the specific amounts ranged from 120,000 to 13,787,000 yuan. The hospitals’ financial assistance income data were not completely normally distributed before and after implementation, so a Mann–Whitney Test was conducted to compare the difference. The result turned out to be significant (u = 116, *p* = 0.023 < 0.05). After implementation, the average increase was 1,665,200 yuan (79.94 %). Fifteen hospitals exhibited very different degrees of growth, and eight hospitals increased by more than 100 %. However, income from financial subsidies at four of the hospitals fell, and the maximum decrease was 60.62 %. One hospital exhibited almost no change in financial assistance income after implementation.

1.2.2 Changes in the total cost of medical insurance compensation before and after implementation

The total costs of medical insurance compensation at the county hospitals before and after the implementation of the Essential Medicines System were compared to understand the changes in the medical insurance funds Table 3.

**Table 3:** Changes in the total costs of health insurance compensation at the county hospitals before and after the implementation of the Essential Medicines System

County-level hospitals	Before implementation (10 thousand yuan)	After implementation (10 thousand yuan)	Difference (10 thousand yuan)	Range of increases (%)
A	1144.53	1064.15	−80.38	−7.02 %
B	2974.36	3648.45	674.09	22.66 %
C	3479.5	3355	−124.5	−3.58 %
D	1061.07	1497.58	436.51	41.14 %
E	4998	7112	2114	42.30 %
F	1121.55	1569.86	448.31	39.97 %
G	153.37	158.53	5.16	3.36 %
H	2882.38	3324.14	441.76	15.33 %
I	916.09	1118.17	202.08	22.06 %
J	2489.11	2562.42	73.31	2.95 %
K	892.5	1023.32	130.82	14.66 %
L	2189.44	3034.28	844.84	38.59 %
M	559.94	1294.3	734.36	131.15 %
N	1310.37	1104.16	−206.21	−15.74 %
O	1300.5	1415	114.5	8.80 %
P	794.26	1095.61	301.35	37.94 %
Q	782.25	1305.7	523.45	66.92 %
R	1057.42	1450.62	393.2	37.18 %
S	1111.73	1230.73	119	10.70 %
T	2760	2017	−743	−26.92 %
Average	1698.92	2019.05	320.13	18.84 %

Before implementation, the total cost of health insurance compensation averaged 16,989,200 yuan, and specific compensation funds ranged from 1,533,700 to 49,980,000 yuan. After implementation, the total cost of medical insurance compensation averaged 20,190,500 yuan, and ranged from 1,585,300, to 71,120,000 yuan. The two groups of data were not normally distributed, and comparison with a Mann–Whitney Test revealed that the difference was of no significance (u = 153, *p* = 0.204 > 0.05). The average cost of medical insurance compensation had gone up by 3,201,300 yuan (18.84 %) after implementation. Only four hospitals witnessed larger growth rates which were more than 40 %, while others experienced small fluctuations, mostly with an increase or decrease rate between 3 % and 30 %.

1.3 Effects on the operation of the hospitals of implementing the policy (i.e., the hospitals’ incomes, service amounts and fees)
1.3.1 Changes in income from medicine sales and proportion of income derived from medicines at the county hospitals before and after implementation

After implementation, the average income from medicines changed from 23,859,400 yuan to 24,703,400 yuan. Overall, the difference was small, and the increase was only 844,000 yuan (3.54 %). The index values of each hospital were similar before and after implementation, but there were still slight fluctuations post-implementation. Eight hospitals exhibited declines in income from medicines after implementation, and this decline was most obvious in hospital F, which experienced a 30.76 % decline. The other 12 hospitals experienced increases, and hospital M experienced the largest increase of 67.1 %.

We also analyzed the proportion of income derived from medicines and the results are displayed in Table 4.

**Table 4:** Changes in proportions of income derived from medicines at the county hospitals before and after the implementation of the Essential Medicines System

County-level hospitals	Before implementation (%)	After implementation (%)	Difference (%)	Range of increases (%)
A	41.61	37.12	−4.49	−10.79
B	46.21	36.29	−9.92	−21.47
C	44.39	40.91	−3.48	−7.84
D	33.65	31.39	−2.26	−6.72
E	48.31	35.67	−12.64	−26.16
F	49.66	36.29	−13.37	−26.92
G	49.86	42.02	−7.84	15.72
H	50.09	42.57	−7.52	−15.01
I	49.98	42.43	−7.55	−15.11
J	51.45	49.02	−2.43	−4.72
K	46.21	42.22	−3.99	−8.63
L	50.89	44.73	−6.16	−12.1
M	59.73	55.95	−3.78	−6.33
N	48.82	42.59	−6.23	−12.76
O	56.72	48.97	−7.75	−13.66
P	72.16	77.07	4.91	6.8
Q	44.36	44.79	0.43	0.97
R	66.64	65.11	−1.53	2.3
S	46.21	47.75	1.54	3.33
T	38.54	32.57	−5.97	15.49
Average	49.77	44.77	−5	10.05

Before implementation, the average proportion of income derived from medicines was 49.77 % with a range of 33.65 % to 72.16 %. After implementation, the average proportion of income derived from medicines was 44.77 %, which represented a reduction of 5 % (10.05 %). These proportions ranged from 31.39 % to 77.07 %. The proportion of income derived from medicines declined in 17 hospitals, and hospital F exhibited the largest decline of 26.92 %. Only hospitals P and S exhibited slight increases of 6.8 % and 3.33 %, respectively. One hospital exhibited almost no change.

1.3.2 Changes in the amounts of service before and after implementation

The amounts of service of the hospitals were measured in terms of inpatient, outpatient and emergency visits. Before implementation, the average number of outpatient and emergency visits was 98,460. After implementation, this number was 120,547, which represented an increase of 22,087 (22.43 %). This number increased in all but one of the hospitals, and the largest increase was 83.77 %. Only hospital N experienced a decline of 4.85 %.

Before implementation, the average number of inpatient visits was 8,614. After implementation, this number was 10,230, which represented an increase of 1,616 (18.76 %). The number of inpatient visits increased in 17 hospitals, and the highest increase was 67.61 %. Inpatient visits in three hospitals were reduced after implementation; hospitals G, F and N experienced reductions of 23.46 %, 9.98 % and 5.83 %, respectively.

1.3.3 Changes in service fees before and after implementation

Before implementation, the average outpatient per-visit fee was 170.28 yuan, and these fees ranged from 89.42 to 314 yuan. After implementation, the average fee was 159.49 yuan, and no large differences before and after implementation were observed. Only two hospitals exhibited obvious declines; fees in hospital I were reduced by 46.62 %, and fees in hospital K were reduced by 37.97 %. Hospital T’s costs increased by 25.29 %, but the changes in the other hospitals were small.

Before implementation, the average per-visit hospitalization fee was 4,182.84 yuan, and these fees ranged from 3,109.6 to 5,665.5 yuan. After implementation, the average per-visit hospitalization fee was 3,695.44 yuan, and these fees ranged from 2,358.91 to 4,781 yuan. These two sets of data were normally distributed, and a two-sample paired *t* test revealed a significant difference (t = 4.392, *p* = 0.000 < 0.05). After implementation, the per-visit hospitalization fee was 487.41 yuan lower than before, and the average decline was 11.65 %. Fifteen of the hospitals’ costs declined to different degrees, and hospital B exhibited the largest decline of 29.2 %. The specific results are illustrated in Table 5.

**Table 5:** Changes in the per-visit hospitalization fees at the county hospitals before and after the implementation of the Essential Medicines System

County-level hospitals	Before implementation (yuan)	After implementation (yuan)	Difference (yuan)	Range of increases (%)
A	5665.5	4781	−884.5	−15.61
B	4679.95	3313.4	−1366.55	−29.2
C	4672	3323	−1349	−28.87
D	3411.93	3453.9	41.97	1.23
E	4645.5	3953	−692.5	−14.91
F	4183.4	3340.2	−843.2	−20.16
G	3109.6	3137.2	27.6	0.89
H	4801.4	4343.62	−457.78	−9.53
I	4149.5	4573.5	424	10.22
J	5370.68	4590.9	−779.78	−14.52
K	4060.57	3312.1	−748.47	−18.43
L	4477.5	4223	−254.5	−5.68
M	3590	2871	−719	−20.03
N	3908	3586	−322	−8.24
O	2867.22	2358.91	−508.31	−17.73
P	4163.37	3463.78	−699.59	−16.8
Q	3126.96	3528.46	401.5	12.84
R	3665.28	3697.79	32.51	00.89
S	4819.5	4116	−703.5	−14.6
T	4289	3942	−347	−8.09
Average	4182.84	3695.44	−487.41	−11.65

2. Analysis of the factors that influenced the implementation outcomes of the Essential Medicines System

The service fees directly reflect the level of expense control by the hospital, have a close relation with the vital interests of the masses and can be the easiest to perceive. Reducing service fees was precisely one of the key purposes of implementing the Essential Medicines System. Therefore, we focused on changes in the service fees, which were used as direct and effective measurement instruments of the system outcomes, and explored the potential factors that caused the transformation. Because the differences in per-visit outpatient fees were not statistically significant, the per-visit hospitalization fees were analyzed.

We first identified the conceivable influential factors, which included a total of seven variables: financial assistance income, total medical insurance compensation, income from medicines while hospitalized, hospital examination income, examination charges for large scale medical equipment, the actual number of types of stocked essential medicines, and the proportion of sales that were essential medicines. Next, we conducted a single factor linear regression analysis. The results are illustrated in Table 6.

**Table 6:** Linear regression analysis of the factors that affected per-visit hospitalization fees

Independent variables	Depedent variable	Sig.
Financial assistance income	Per-visit hospitalization fees	0.029*
Total medical insurance compensation		0.086
Income from medicines while hospitalized		0.006*
Income from hospital examinations		0.906
Examination charge of large scale medical equipment		0.145
Actual number of varieties of essential medicines		0.015*
Proportion of actual sales of medicines that were essential medicines		0.046*

*Means *p* < 0.05

The results revealed that three elements, medical insurance compensation, hospital examination income and examination charges for large-scale medical equipment were statistically of no significance to the changes of per-visit hospitalization fees. In contrast, the remaining four variables seemed to be associated with per-visit hospitalization fees to some degree, with financial assistance revenue (*p* = 0.029 < 0.05), income from medicines during hospitalization (*p* = 0.006 < 0.05), the actual number of types of essential medicines (*p* = 0.015 < 0.05) and the proportion of sales that were essential medicines (*p* = 0.046 < 0.05), respectively.

Based on the single factor analysis, multiple linear regression analysis was used to further explore the causal relationships between the variables. The per-visit hospitalization fees was still used as the dependent variable. Financial assistance revenue, income from medicines during hospitalization, the actual number of types of essential medicines and the proportion of sales that were essential medicines, all proven to be significant in the univariate analysis, were considered as variables in the second round. The regression model produced F = 5.502 and *p* = 0.002 and was thus statistically significant. The results are shown in Table 7.

**Table 7:** Multiple linear regression analysis of the factors that affected per-visit hospitalization fees

Model	Unstandardized coefficients		Standardized coefficients	t	Sig.
	B	Std. error	Beta		
(Constant)	3879.162	331.453		11.704	0.000
Financial assistance revenue	0.905	0.355	0.347	2.550	0.015*
Hospitalization medicine income	0.239	0.113	0.302	2.122	0.041*
Number of types of essential medicines	−2.008	1.850	−0.239	−1.085	0.285
Proportion of actual sales that were essential medicines	−15.216	22.174	−0.144	−0.686	0.497

*Means *p* < 0.05

Seen from the results, only two variables, financial assistance revenues and income from medicines during hospitalization, should be included in the regression equation. The regression coefficient for the financial assistance revenue was r = 0.347 (*p* = 0.015 < 0.05), and the regression coefficient for the income from medicines during hospitalization was r = 0.302 (*p* = 0.041 < 0.05). Thus, increases in income from medicines during hospitalization led to increases in per-visit hospitalization costs. Unexpectedly, greater financial assistance revenue also led to higher average per-visit hospitalization costs.

## Discussion

1. The guiding principle of the National Essential Medicines List remains to be reinforced, and specific lists for county hospitals should be developed.

We learned from the interviews that, currently, the drug directory for the county-level public hospitals in Anhui Province seems to be in some disorder and this may weakened the leading position and the guiding principle of the National Essential Medicines List. We therefore suggest the abolishment of province-level supplement directories of essential medicines, which are not convenient for unified management and standard medication. Additionally, due to the large patient populations encountered by county hospitals, these hospitals may have to deal with various diseases and a wide variety of drugs are in need. Successful experiences in Delhi [12] and South Africa [13] have shown that the development of different levels of the List can be effective on the policy improvement. Thus, it is worthwhile to develop the specialized Essential Medicines List for county hospitals.

2. Supervision was required for the implementation process; the supply mechanism for the essential medicines requires further improvement.

Although the average stock levels and sales of essential medicines at the county hospitals increased after implementation, these sales still did not reach the required standards, which was issued in the previous provincial documents, claiming that the types of essential medicines that are used by secondary-level general hospitals should not be less than 95 % of the total number of essential medicines and that the proportion of total sales that are essential medicines should not be less than 30 %. This finding revealed a serious lack of policy execution strength. Without powerful supervision, the enthusiasm of the hospitals could not be fully mobilized. Follow-up investigations and timely feedback are needed.

Serious problems existed in the supply chain, which resulted in untimely and incomplete distributions of essential medicines. Previous studies [14, 15] have shown that uncertainties about national policy, low profit levels for essential medicines and the internal problems of distribution companies lead to problems in the distribution chains. Clear and adequate supplies resulted in a significant higher usage rate of essential medicines [16]. In response, state and local government could directly specify that the manufacturers and distributors of essential medicines establish a fast and efficient supply chain.

3. The compensation mechanism was far from sound and adequate, and the leverage of the health insurance policy was not obvious.

Financial assistance from the government is considered as the most essential factor for the System implementation. Without sufficient investment, hospitals will struggle to survive [17]. Currently, as required by the policy, 25 % of the loss of income for the county public hospitals in Anhui is subsidized by the provincial government, and another 75 % is subsidized by the examination fees that are charged by the hospital. Some of the county hospitals reported that since the implementation, their revenues had decreased significantly and that the additional financial assistance and examination fees were not enough to compensate for this decrease. As solutions, various compensation methods and flexible policies, such as performance grants, should be made available. Moreover, medical services price adjustment rights could be unified by municipal departments and the progress of implementation.

After implementation, when payments for the examination fees were accepted by the medical insurance funds, the policy resulted in a significant increase in the burden of medical insurance. However, the total capital in the system increased very little. The medical insurance compensation alone is not responsible for the promotion of the smooth operation of the Essential Medicines System. Thus, rather than relying on increasing compensation, the leverage role of medical insurance should be further developed. Multiple payment methods ought to be implemented in our country because the payment methods in developed countries, such as Australia and the UK, are based on the type of disease or the effect [18], and Australia had also set up copayments and safety nets for essential medicines for outpatients [19–21].

4. The policy was partially successful because of the reduction in the proportion of income from medicines and the average inpatient fees of the county hospitals.

The average proportion of income from medicines, which is an important indicator of the comprehensive management capabilities of hospitals, was reduced by 5 % after implementation. This reduction may have been due to the levels of rational drug use by the hospitals, which were improved, and this finding partially suggests that the implementation of the Essential Medicines System was successful. However, the proportion of income from medicines remained as high as 44.77 %, which indicates that this remains one of the most important sources of income for the hospitals.

After implementation, outpatient and emergency visits and inpatient visits increased likely due to the reduction in medicine prices and the increase in the reimbursement ratio, which attracted more patients. These results are consistent with another Chinese report in which outpatient and inpatient visits to hospitals increased by 0.74 % and 16.39 %, respectively [22]. To some extent, these increases alleviated the pressure on to the masses to see doctors.

A survey of three provinces in China found that the costs of outpatient and inpatient visits decreased by 4.9–14.6 % and 7.4–13.4 %, respectively, after the implementation of the Essential Medicines System [23]. However, our survey found that the per-visit outpatient fees were not significantly different after implementation. In contrast to the study of Wang Jincai, which found that the per-visit outpatient and inpatient fees increased by 18.46 % and 3.74 % [22], respectively, the per-visit hospitalization fees were found to be reduced by an average of 11.65 % after implementation in our research, which indicates that the issue of expensive treatment is likely being successfully addressed.

5. The actual usage of the subsidies in the hospitals should be given more attention.

In the univariate analysis of the potential influential factors, the stock levels of essential medicines and the proportions of sales that were essential medicines were both found to have an impact on the per-visit hospitalization fees. Theoretically, higher proportions of stock levels and usage of essential medicines should lead to lower per-visit hospitalization costs. However, the effects of these two variables were eliminated in the multivariate analysis. It may be that the effects of these two factors on the reduction in the medical fees of the hospitals were insufficient at the present and should be further strengthened.

Our analyses resulted in the surprising conclusion that the per-visit hospitalization fees increased with increasing financial assistance incomes, which was the opposite of the intention of the policy. This finding suggests that the specific usage of assistance income by the hospitals was not clear. Indeed, the subsidies should be provided directly to doctors. Only by guaranteeing doctors’ incomes and mobilizing their motivation can we avoid high profits on prescription medicines. Therefore, after the establishment of a more sound hospital compensation mechanism, more attention should be given to the use of compensation funds. We suggest specifying the proportion of personnel expenditures in the hospitals for prevention of blind expansion and reduction on the staff remuneration.

## Conclusions

In conclusion, our results showed that the proportion of income derived from medicines and per-visit inpatient fees in the survey county hospitals both went down than before. These results suggest that implementation of the Essential Medicines System do have a beneficial role in the reduction of the drug fees and further alleviates the burden of the masses. However, without sound and perfect supporting measures, the Essential Medicines System is bound to encounter lots of barriers in its exploratory stage. Much effort should be made to the redesign of the compensation mechanism, mainly including the government and the medical insurance compensation, emphasizing on both the fairness and the rationality of the compensation. Moreover, to gain insight in long-term effects of the Essential Medicines System, a follow-up survey should be conducted in the future.

### Limitations

The implementation period of the Essential Medicines System in the county-level hospitals in China was short, and understanding of the policy implications varied widely. Simultaneously, the supplementary measures were not synchronously established or perfect. On this circumstance, our study can only be exploratory.

There might be some omissions in the indicators since we used self-designed questionnaires for investigation. Although some quality control measures were taken during this investigation, some individual error may inevitably exist in our study. While the words frequency analysis were used for the qualitative data, the accuracy and sufficiency were somewhat limited by the small sample size in the survey and analysis. Additionally, our investigation included only 20 county public hospitals in Anhui. Therefore the results would not be entirely applicable to other areas of China. The implementation outcomes of the Essential Medicines System in different areas should be both horizontally and vertically compared and analyzed in depth.
